# Unprotected Youth Workers in US Agriculture

**DOI:** 10.3389/fpubh.2023.1064143

**Published:** 2023-05-30

**Authors:** Lisa Iannacci-Manasia

**Affiliations:** ^1^School of Nursing, Columbia University, New York, NY, United States; ^2^School of Nursing, Molloy University, Rockville Centre, NY, United States; ^3^Department of the Sciences of Public Health, Nursing & Pediatrics, University of Turin, Piedmont, Italy

**Keywords:** child labor, agriculture, social determinants, legal epidemiology, health outcomes, policy

## 1. Introduction

Agricultural work is the most hazardous and grueling area of employment open to youth in the United States of America (US) (1–3). Farming is the only work setting where youth of all ages are legally permitted to work across all fifty states. A 2018 US Government Accountability Office study found that more than half of work-related youth fatalities occurred among youth working in agriculture (4). For most industries, the federal Fair Labor Standards Act (FLSA) sets minimum standards of protection for hired workers, which individual states can exceed if they so choose. Agriculture, however, is exempted from provisions of the FLSA due to outdated exceptions from when the FLSA was enacted in 1938 (5). As a result, youth working in US agriculture are not as legally protected as youth working in other industries.

### 1.1. Lax federal and state child labor laws in agriculture

For all US industries excluding agriculture, the basic minimum age for employment is 16, employment under 14 is prohibited and 14- to 15-year-old youth can only work limited hours in certain occupations (6, 7). Under federal law, hired workers under 12 years old may legally work in agriculture with no limits on hours worked per day or days worked per week (8). The FLSA agricultural exemptions permit youth working on farms to do work that the US Department of Labor has deemed “particularly hazardous … or detrimental to their health or wellbeing” at younger ages than working youth in other industries (9). Youth workers in agriculture can engage in these hazardous occupations and perform dangerous tasks at 16 years old while workers in all other industries must be 18 years old to do similarly hazardous work (6, 10). Without the legal redress afforded to all other working youth, those working on farms are not guaranteed overtime pay, leading employers to give them longer hours. These young, hired workers in agriculture experience interruptions in their education and risk serious preventable injury, illness, or death from exposure to heat, chemicals, hazardous machinery, and environmental perils, all of which can cause deleterious health effects leading to life-long, negative consequences for their health and wellbeing.

As a result of these lax federal standards, the fifty individual states have significant discretion to provide—or fail to provide—legal protections for young, hired workers in agriculture. Laws can vary from state to state, but most states' laws do not set more stringent standards than the FLSA does for young, hired workers in agriculture—and those that do are still more permissive than the FLSA is for other industries. Just a taste: 21 states permit youth of any age to work in agriculture, and age minimums in the other 29 states vary from 10 to 14 years old; 26 states set no limit on the maximum number of hours hired youth can work each day; 35 states allow hired youth to work seven days a week, while the other 15 states only require a single day off; and 23 states have no laws prohibiting hired youth from working nights. Levels and severity of enforcement vary even more between states: five states have no fines for violations, while, in the other 45 states, fines range from $500 to $10,000; furthermore, 34 states categorize violations as criminal acts, with 32 categorizing violations as misdemeanors, i.e., a criminal offense punishable with imprisonment for up to 364 days, and only two states categorizing them more severely as felonies, i.e., criminal offenses punishable with imprisonment for 365 days or longer (11, 12) (Table 1).

**Table 1:** Summary of the 6 U.S. child labor law features for the 50 United States.

**1. Minimum working age**	**21 U.S. states have no minimum age**	**29 U.S. states: vary from 10–14 years**
**2. Maximum hours per day**	26 U.S. states set no limit	24 U.S. states: maximum daily hours vary by age & seasons
**3. Maximum days per week**	35 U.S. states allow 7 days	15 U.S. states allow 6 days
**4. Prohibited night work**	23 U.S. states have no laws	27 U.S. states have laws
**5. Monetary fines for violation**	5 U.S. states have no fines	45 U.S. states have fines ranging $500–$10,000
**6. Criminal penalties for violation**	16 U.S. states do not make it a crime	2 U.S. states categorize as felony, 32 categorize as misdemeanor

### 1.2. Negative health consequences for young, hired workers in agriculture

US laws and policies currently governing child labor leave young hired workers in agriculture unprotected. There is no official nationwide data for children younger than 18 years old who work in agriculture. In a report released in October 2021, the Child Labor Coalition and Lawyers for Good Government estimated that, in the US, 330,000 children younger than 16 years old—including over 80,000 children younger than 10 years old—are hired workers in agriculture (12). The most recent Childhood Agricultural Injury Survey reported approximately 600,000 youth younger than 18 years old (approximately 15% younger than 10 years old, 46% between 10 and 15 years old, and 39% either 16 or 17 years old) working on farms across the US (13). In addition to educational disruption, these young agricultural workers are exposed to many occupational health risks with both short- and long-term consequences, including respiratory disease, neurotoxicity, pesticide toxicity, heat illness, musculoskeletal injuries, traumatic injuries, dermatological injuries, discrimination, and sexual harassment. Youth working on US tobacco farms incur the added risk of exposure to nicotine, a known neurotoxin. These risks are severe and widespread (13–44). Approximately 33 children are injured daily in agriculture (45). On average, one child dies in an agriculture-related incident every 3 days (2). Since 2000, young, hired workers have been killed in agriculture-related incidents in at least 49 states. From 2001 through 2015, 48% of all fatal injuries to young, hired workers occurred in agriculture (3).

But these statistics are all lower-bound estimates of the true figures. Researchers estimate that over 90% of young, hired workers in agriculture are persons of color, typically seasonal migrant Latinx. These youth often work in agricultural settings that either elude or are not subject to the surveillance and data collection procedures of the federal Bureau of Labor Statistics and the National Institute for Occupational Safety and Health; substantial numbers of young, hired workers in agriculture will therefore not be included in the best-available federally supplied data (46).

### 1.3. US law does not align with international standards

The outdated US agriculture child labor policy and legal regulatory framework is weak and compromising. It does not align with international labor law and global measures to protect the health and safety of children. The International Labor Organization (ILO) as well as other advocates for working children worldwide including the United Nations Children's Fund and Convention on the Rights of the Child (CRC) establish policy and sustain efforts to eradicate employment that compromises children's opportunities for good health, education, and future potential. The CRC, which the US signed, defines a child as a person under the age of 18 (47). Article 32 states that children are to be protected from economic exploitation and from work that is likely to be hazardous, to interfere with the child's education, or to be harmful to the child's health or physical, mental, spiritual, moral or social development (47). The ILO's Convention No. 138 on Minimum Ages to Employment and Work provides the normative rule of international law and defines unlawful child labor as involving any individual younger than 18 years old doing hazardous work, any individual younger than 15 years old doing any other work other than light work, or any individual younger than 13 years old doing even light work (48). This definition was expanded by ILO Convention No. 182, ratified by the US, functionally defining child labor as work that is inappropriate for a child's age, that prevents a child from benefiting from compulsory education, or that is likely to harm a child's health, safety or morals and hinder his or her development and future livelihood (49, 50). The ILO has strongly urged the US government on multiple occasions to address the gaps compromising the health and wellbeing of youth working in US agriculture (50).

## 2. Discussion

There is much that can and should be done to help these youth. Researchers and academics can use the methodologies of legal epidemiology—the scientific study and deployment of law as a factor in the causation, distribution and prevention of disease and injury in a population—to better inform much-needed changes in policy (51–53). By considering child labor policy and law—as well as workplace environments they create—as social determinants of health, researchers can evaluate differences between state laws regulating young, hired workers in agriculture as a potential contributing factor to health outcomes for these youth. There are large gaps, both in epidemiological data-collection and in our current understanding of the causal links between state child labor laws and injuries and fatalities among young, hired workers in agriculture. Filling in these gaps is crucial for effective changes in policy: policy makers will not only be better informed of the magnitude of the issue but also better positioned to implement more effective changes.

At the very least, however, the outdated agricultural loopholes in the FLSA must be closed. At the federal level, the proposed Children's Act for Responsible Employment, also known as the CARE Act, would do just that: among other things, it would raise the minimum age for especially hazardous work from 16 to 18 years old and prohibit any agricultural work, except on family farms, by anyone younger than 14 years old (53–59). The CARE Act was originally proposed in Congress in 2009 and, despite being reintroduced several times, it has regrettably still not been passed (54–60). Another important legislative attempt to target US tobacco farms specifically is the Children Don't Belong on Tobacco Farms Act, which would prohibit employment of youth younger than 18 years old in tobacco-related agriculture. The bill was first introduced in Congress in 2015 and, though it has also been repeatedly reintroduced, has not been passed (61). But states do not need to wait for Congress to act—they can change their own laws at any time to bring child labor protections for agriculture in line with those for other industries. Whether change comes from the federal level or individual states, it is clear that change is needed. Young hired workers in agriculture are among the most vulnerable and marginalized populations in the US, and they are in dire need of legal protection.

## Author contributions

The author confirms being the sole contributor of this work and has approved it for publication.
